# A Dual-Mode Neural Amplifier Array for Biopotential and FSCV-Based Neurochemical Measurements

**DOI:** 10.3390/bios16090466

**Published:** 2026-08-26

**Authors:** Matthew A. Crocker, Kevin A. White, Mahdieh Darroudi, Vishnu Saket S. Bapanapalli, Charles S. Lipscomb, Benjamin S. John, Brian N. Kim

**Affiliations:** Department of Bioengineering, University of Texas at Dallas, Richardson, TX 75080, USA; matthew.crocker@utdallas.edu (M.A.C.); kevin.white@utdallas.edu (K.A.W.); vishnusaket.bapanapalli@utdallas.edu (V.S.S.B.); charles.lipscomb@utdallas.edu (C.S.L.); benjamin.john@utdallas.edu (B.S.J.)

**Keywords:** neurochemical, biopotential, simultaneous recording, complementary metal-oxide semiconductor, CMOS, fast-scan cyclic voltammetry, FSCV, neural interface, neurotransmitter

## Abstract

The simultaneous measurement of biopotential and neurochemical signals provides a comprehensive view of the brain. Yet, most neural interfaces record solely biopotential or neurochemical activity. This work presents a complementary metal-oxide-semiconductor (CMOS) analog front-end (AFE) chip that integrates 32 biopotential amplifiers and 32 neurochemical amplifiers for parallel recording from 64 electrodes. The biopotential amplifier is a two-stage design providing a gain of 57.1 dB, a bandwidth of 0.4 Hz–6.2 kHz, and 6.7 µV_RMS_ input-referred noise (20 kHz sampling rate). The neurochemical amplifier is a rail-to-rail folded-cascode operational amplifier with selectable transimpedance gain (91.9 kΩ to 851.9 kΩ), a dynamic range of ±15 μA to ±2 μA, respectively, a bandwidth of 12.6 kHz, and input-referred noise as low as 46.3 pA_RMS_ (20 kHz sampling rate). The neurochemical amplifiers are designed for fast-scan cyclic voltammetry (FSCV) measurements. I/O complexity is minimized using a time-division multiplexing scheme for readout, enabling straightforward scalability. The chip is fabricated using a 0.35-µm CMOS process and occupies a 3.0 × 8.3 mm^2^ area. In vitro recordings of catecholamines and neural spikes validate the chip’s function. The chip enables scalable, low-noise, bimodal neural recording, supporting investigations into the dynamics between neuronal biopotential activity and neurochemical signaling.

## 1. Introduction

Technologies for recording neuronal action potentials and local field potentials have improved substantially in recent years, improving both spatial and temporal resolution. In contrast, characterizing the heterogeneous spatiotemporal distribution of neurotransmitters, such as catecholamines like dopamine, remains a challenge. Catecholamines act as key modulators of motor control, motivation, and reward-related behaviors [1,2,3,4,5]. Characterizing the heterogeneous spatiotemporal distribution of catecholamines provides insight into their function. Existing approaches for analyzing neurotransmitters in the brain include microdialysis, functional magnetic resonance imaging, and assorted electrochemical techniques. Microdialysis extracts cerebrospinal fluid directly from brain tissue, but delays between sampling and analysis prevent real-time monitoring. Functional magnetic resonance imaging enables high-resolution mapping of neurotransmitter dynamics and has been applied to the analysis of catecholamines [6,7,8,9]. However, its sensitivity is insufficient for resolving sub-micromolar fluctuations [8,9]. Fast-scan cyclic voltammetry (FSCV), an electrochemical technique, is widely used to measure catecholamines in vivo [10,11,12,13,14,15,16,17,18,19,20,21]. In FSCV, a triangular voltage waveform (typically ranging from −0.4 to 1.3 V at 100–400 V/s) drives redox reactions at the electrode surface. The amplitude of the measured redox signals, the oxidation current during the positive ramp and reduction current during the negative ramp, directly relate to the concentration of redox molecules at the electrode surface. Additionally, FSCV provides chemical specificity for measurements; the potential the maximum amplitude of the oxidative and reductive signals occur can be used as identifying features specific to the measured chemical. Redox signal recording using FSCV for in vivo measurements can be on the scale of nanoamperes [21]. Using FSCV, a relatively large ‘background’ current is recorded due to the electrode’s double-layer capacitance (on the scale of microamperes). To isolate the small faradaic signals from redox reactions, background subtraction is used to remove the baseline background signal. Additional techniques, like modifying FSCV’s waveform, can be employed to optimize measurements for specific targeted chemicals [22,23].

To capture the spatiotemporal heterogeneity of neurochemical signals using FSCV, high-density microelectrode arrays (MEAs) can be used. Individual electrodes can localize redox reactions to individual sites, thus enabling spatially resolved tracking of neurochemical dynamics. Each microelectrode requires signal amplification, and compact circuitry is essential for in vivo applications. Complementary metal-oxide-semiconductor (CMOS) technology addresses these requirements by enabling dense integration of amplifier arrays in small footprints, supporting high-throughput measurements.

Although this work focuses on central nervous system applications, the same multimodal approach can be applied to the peripheral nervous system. For example, FSCV has been used to measure real-time norepinephrine release in the splenic neuroimmune circuit [24]. Combining neurotransmitter measurements with biopotential recordings could provide additional information about autonomic regulation and neuroimmune signaling. These applications further highlight the need for scalable, low-noise front ends that can support diverse electrode technologies.

FSCV-based technologies enable both in vitro and in vivo neurochemical measurements [25,26,27,28,29,30,31]. TinyFSCV supports single-channel FSCV with scan rates up to 400 V/s and an input range of ±115 µA using discrete components, at the cost of a large 40 mm × 24 mm footprint [25]. IC implementations reduce area while enhancing noise performance, such as a single-channel cyclic voltammetry chip supporting 0.008–400 V/s scan rates with 25 nA_RMS_ of noise with a sampling rate of 50 kS/s in a 1.6 mm × 2.0 mm footprint [27]. A 128-channel array achieves 220 pA_RMS_ noise over 20 kHz of bandwidth with a ±5 µA dynamic range [28]. Other designs include an analog front-end (AFE) chip with 39.2 pA_RMS_ of noise (5 kHz of bandwidth) [29]. A wireless single-channel system achieves 92 pA_RMS_ noise over 2 kHz of bandwidth with a ±430 nA dynamic range [30].

High-density FSCV recordings require a neurochemical front-end that combines high gain with a dynamic range able to accommodate the large background currents at the electrode-electrolyte interface. The current’s magnitude depends on electrode material, geometry, surface treatment, and scan rate. A typical 7 µm carbon fiber electrode produces sub-µA background currents [32,33]. A similarly sized planar gold electrode can produce 0.5–2 µA [34,35,36]. While a coated gold electrode can reach 2+ µA. The background charging current is approximately proportional to the interfacial capacitance and scan rate, and the total input range needs to accommodate both the background charging current and the faradaic currents from neurotransmitter redox reactions. Changes in electrode design and surface modifications can significantly affect the required input range [19,22,37,38,39,40,41]. Selectable gain allows the same front-end to accommodate different electrode configurations while maintaining sensitivity to small redox currents. Different electrodes can therefore be tested while the AFE remains untouched.

Recent CMOS-based devices for in vivo biopotential recording achieve significant advances in channel density, noise performance, and power efficiency [42,43,44,45,46,47,48,49]. A 256-channel integrated circuit (IC) achieves 6.32 µV_RMS_ of noise over a 10 kHz bandwidth with 10-bit ADCs in a compact 1.53 mm^2^ footprint [42]. The Neuropixels 2.0 extends scalability to 384 simultaneously recordable channels and incorporates 14-bit ADCs, while achieving 7.2 µV_RMS_ of noise over a 10 kHz bandwidth [43]. A 512-channel design achieves 4.8 µV_RMS_ of noise over a 300–6000 Hz bandwidth [44]. A 1024-channel IC, which reduces requirements for data throughput by discarding redundant baseline samples, achieves 7.4 µV_RMS_ of noise (300–6000 Hz) in a 36 × 36 µm^2^ footprint, consuming only 268 nW of power per channel [45]. At the extreme end of parallel recordings, a microwire array demonstrates ~30,000 in vivo recordings with 6.32 µV_RMS_ noise (300–6000 Hz). However, the design requires a large amount of support circuitry, limiting its use to acute head-fixed environments [46,47,48]. Wireless approaches have also emerged, such as a dual-band transmitter achieving 54 Mb/s with 8.0 µV_RMS_ of noise (10–10,000 Hz bandwidth) in a 3.72 × 3.68 mm^2^ area [49].

While the devices that have been referenced demonstrate sufficient capability for in vivo applications, they are limited to either biopotential or neurochemical modalities without concurrent recording capabilities. While it is possible to use a single electrode for multiple modalities, the interleaving of measurements can introduce prohibitively long settling periods after switching between modalities [50]. As a result, we focus on systems that present concurrent modalities using separate electrodes for recording. A limited set of integrated devices enables simultaneous non-interleaved multimodal sensing [51,52,53,54,55,56,57]. A two-channel AFE chip supporting both local field potential recording and FSCV achieves 5 µV_RMS_ of noise (1-200 Hz bandwidth) for the biopotential channel and 50.2 pA_RMS_ noise (100 kHz bandwidth) with a gain of 100 kω for the neurochemical channel [51]. A 200-channel MEA demonstrates dual-mode operation, with neurochemical sensing (93 pA_RMS_ of noise, 10 kHz of bandwidth) and biopotential measurements (4.07 µV_RMS_ of noise, 1–100 kHz of bandwidth), though the neurochemical input range is restricted to ±50 nA [52]. Our prior work is a 512-channel electrophysiology and electrochemical dual-mode CMOS chip with 256 electrophysiology amplifiers, 256 electrochemical amplifiers, and an on-chip microelectrode array of 512 electrodes, designed for in vitro studies of action potential propagation and synaptic neurotransmitter secretion across neuronal networks. This work splits the amplifiers evenly between modalities, achieving 24.9 µV_RMS_ of noise (0.2–10 kHz of bandwidth), and 4.51 pA_RMS_ of noise (10.3 kHz of bandwidth) [57]. Despite these advances, the number of devices supporting large-scale, low-noise, simultaneous multimodal recordings is still extremely limited.

This work presents an AFE IC integrating 32 biopotential and 32 neurochemical amplifiers for future simultaneous electrophysiological and neurochemical recording. The neurochemical front-end provides wide dynamic range, low noise, and selectable gain settings to accommodate microelectrodes with varying capacitances and background currents. Each 32-channel array is time-division multiplexed to a dedicated output for scalable integration.

## 2. A Highly Scalable Neural Amplifier Design

The biopotential and neurochemical amplifier array are fabricated using a 0.35-µm CMOS process. The design balances low-noise integration with high dynamic range capabilities. The array contains 64 neural amplifiers, with 32 allocated to biopotential recording and 32 to neurochemical sensing.

### 2.1. Neurochemical Amplifier

The amplifier is designed for FSCV, which demands detection of large, rapidly varying displacement currents (microampere scale) caused by the electrode-electrolyte double-layer capacitance at the electrode surface. While the simplest solution to accommodate the large current in the measurement is to reduce the gain, this limits the resolution of small redox peaks caused by electroactive molecules such as dopamine. Prior works have adopted a background subtraction strategy to remove the large displacement current [29], however, this approach is not attractive to existing users who rely on the shape of the background current to evaluate the real-time condition of the electrode, which can deteriorate over time due to various fouling mechanisms. Therefore, the best approach is to maximize the dynamic range while maintaining the resolution. During FSCV measurements using microelectrodes, the targeted transimpedance gain is typically between 0.1–1 µA/V (100 kΩ–1 MΩ).

**Figure 1:**
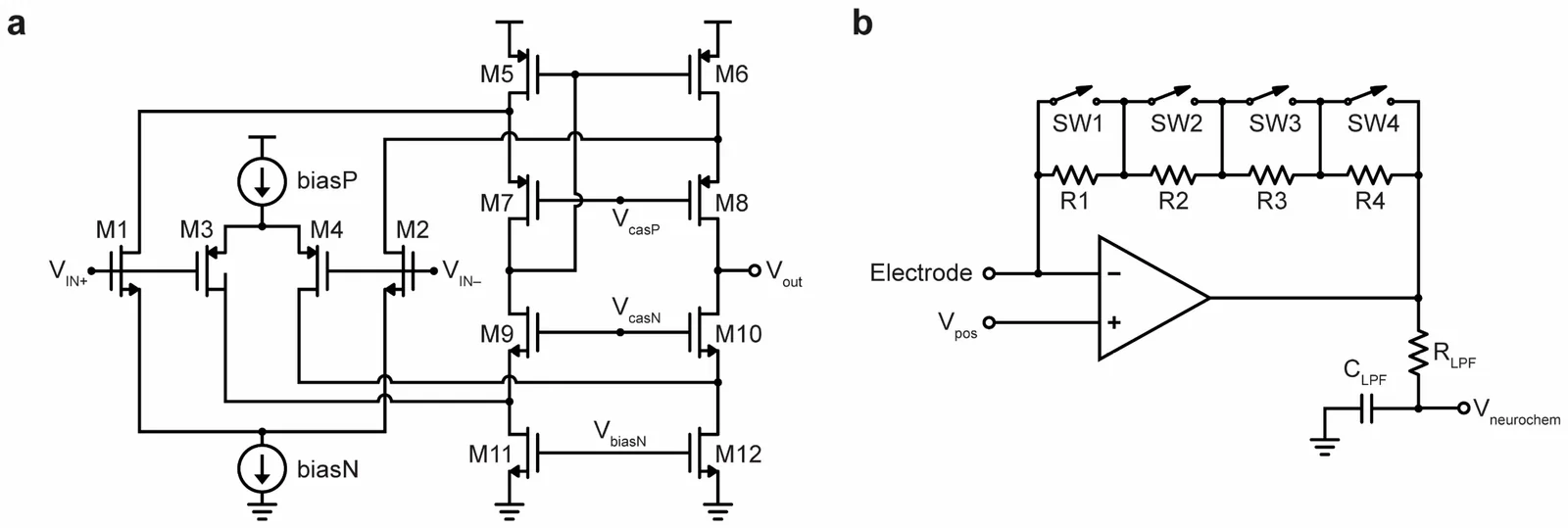
Schematic of the neurochemical amplifier. (**a**) The operational amplifier design of the neurochemical amplifier adapts a rail-to-rail folded-cascode topology. (**b**) The neurochemical amplifier employs a series of resistors with parallel switches to allow variable resistance for the feedback loop. The integrated switches select the variable-gain settings.

The device uses 5 V thick-oxide transistors, with an operational amplifier based on a rail-to-rail folded-cascode design to maximize the recording dynamic range (Figure 1a). The rail-to-rail architecture uses complementary differential pairs: an nMOS differential pair (M1–M2) and a pMOS differential pair (M3–M4). The nMOS differential pair folds into a pMOS cascode (M7–M8), while the pMOS differential pair folds into an nMOS cascode (M9–M10).

The neurochemical amplifier is designed using resistive feedback (Figure 1b) [28,58]. The feedback resistor determines the transimpedance gain. Variable gain is implemented with resistors R1–R4 (50 kΩ, 50 kΩ, 100 kΩ, and 800 kΩ) that are controlled by the switches SW1–SW4. Closing a switch removes the corresponding resistor from the feedback loop, reducing the amplifier gain. For example, closing SW3 and SW4 sets the gain to R1 + R2. With all switches open, the gain is equal to R1 + R2 + R3 + R4.

The RC low-pass filter (R_LPF_, C_LPF_) is used to set the amplifier bandwidth. Each amplifier occupies 200 µm × 600 µm (0.12 mm^2^). The total area the 32 neurochemical amplifiers occupy is 3.84 mm^2^.

During FSCV recordings, it is common to measure large background currents in the range of a few µAs due to the electrode’s double-layer capacitance. The magnitude of the background current is directly related to the material and surface area of the electrode, as well as the scan rate being used. For a typical carbon fiber electrode setup, the background current is below 1 µA [32,33]. However, larger microelectrodes can have background current that is in the range of 10 µA or more [34,35,36]. To accommodate large background currents, the biasing level of the neurochemical amplifier must be chosen carefully. Because the biasing current is determined by an external resistor, the power consumption can be adjusted based on the anticipated background current level. In this experiment, the biasing current (I_biasN_) is set to 28.8 µA to provide sufficient headroom over the anticipated background current (~10–15 µA) by the electrodes used in the later section. A total biasing current of 3 × I_biasN_ (86.4 µA, 432 µW) is used, giving each neurochemical amplifier the ability to handle the broad range of background currents it may face. For a typical CFE producing sub-µA background currents, a ±2 µA dynamic range is sufficient and can be achieved with a lower biasing current, reducing the neurochemical amplifier power consumption to 30 µW.

### 2.2. Biopotential Amplifier

The biopotential amplifier is designed for compact, low-noise, high-performance signal amplification of neural activities, including both local field potentials (LFP) and action potentials (APs).

**Figure 2:**
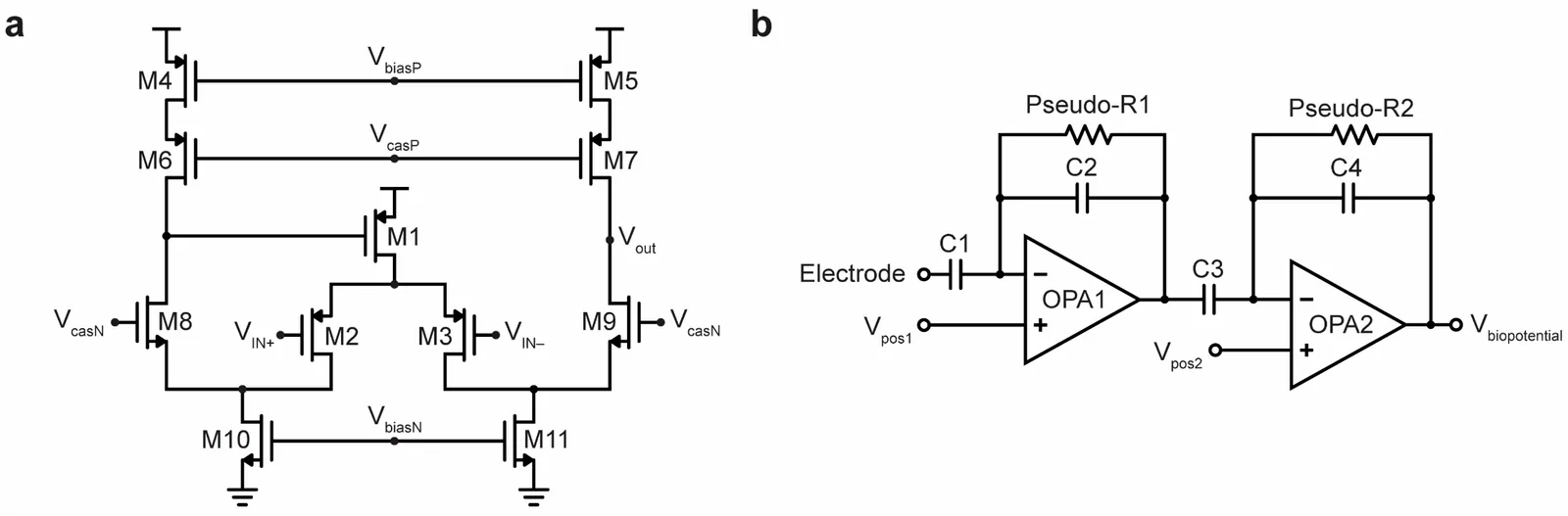
Schematics for the biopotential amplifier. The biopotential amplifier consists of two amplification stages. (**a**) The first stage of the biopotential amplifier is based on a folded-cascode amplifier; it provides a gain of ~150 V/V. (**b**) The second stage of the biopotential amplifier provides a gain of ~5 V/V, producing a total theoretical gain of ~750 V/V. Both stages use pseudo-resistors, based on diode-connected transistors, to provide DC stability at the inverting inputs.

The design employs two stages of amplification. The first stage is based on a low-noise operational amplifier (Figure 2a). It adapts a folded-cascode topology, with M2 and M3 as the differential input pair and M8 and M9 as the cascode stage (Figure 2a). The M1 transistor provides feedback regulation in which the current through M2 and M3 matches the biasing current at M4 [59]. This amplifier is represented as OPA1 (Figure 2b). Stable references (V_pos1_ and V_pos2_) are tied to the non-inverting inputs of both stages and set the DC operating point.

The second stage employs a five-transistor operational amplifier. The inverting inputs of OPA1 and OPA2 are not DC-stable without a resistive path. For DC-stable operation, pseudo-resistors in parallel with C2 and C4 are used [60]. These pseudo-resistors are pMOS transistors with the body and source tied together, and the gate and drain tied together. These provide larger resistance per unit of area than conventional resistors. The total biasing current supplied from M10 and M11 is 19.4 µA, resulting in a power consumption of 97 µW per amplifier. The ratio of the first stage capacitors, C1/C2 (30 pF/200 fF), is multiplied by the ratio of the second stage capacitors, C3/C4 (2 pF/400 fF), to determine the theoretical gain. Unlike the neurochemical amplifier design, the biopotential amplifier is designed to have a fixed gain. The total gain has been set to 750 V/V.

Each biopotential amplifier occupies a 200 µm × 600 µm (0.12 mm^2^) area, equivalent to the neurochemical amplifiers. In total, the 32 biopotential amplifiers occupy 3.84 mm^2^. The array minimizes its footprint while maintaining low noise and high performance, enabling high-density amplifier arrays for neural recording systems.

### 2.3. Multiplexing and CMOS Implementation

The amplifier array output is read out through two separate time-division multiplexing circuits to reduce the total number of outputs and allow greater scalability [28,57,61,62,63,64,65,66]. Time-division multiplexing employs shared lines that enable sequential readouts of amplifier outputs through a single output stage, which significantly reduces wiring complexity which can provide a significant reduction in the footprint size.

**Figure 3:**
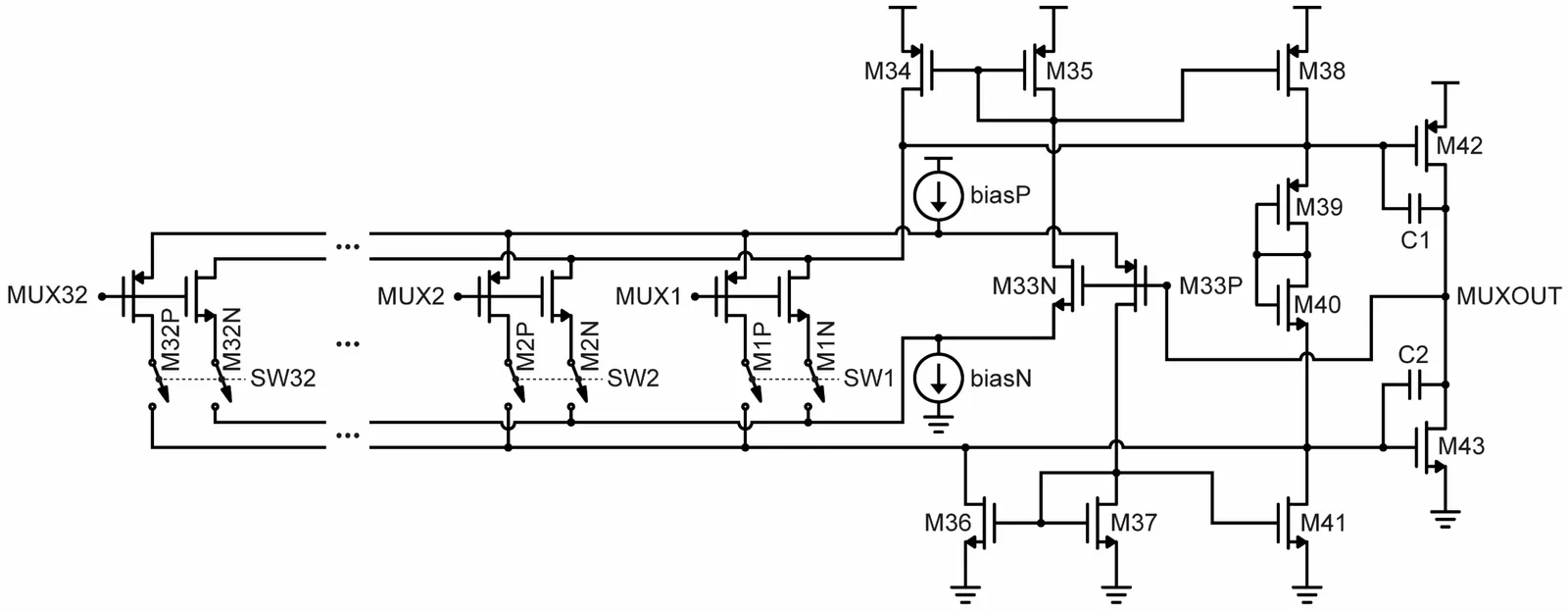
The rail-to-rail multiplexing circuit used for the neurochemical and the biopotential amplifiers. The input transistors, M1N/M1P through M32N/M32P and M33N/M33P, form a unity-gain amplifier in which the non-inverting halves (M1N/M1P–M32N/M32P) share the inverting half of the amplifier, M33N/M33P. The switches (SW1–SW32) sequentially provide the non-inverting halves with mutually exclusive access to the inverting half of the amplifier, which is output at MUXOUT.

**Figure 4:**
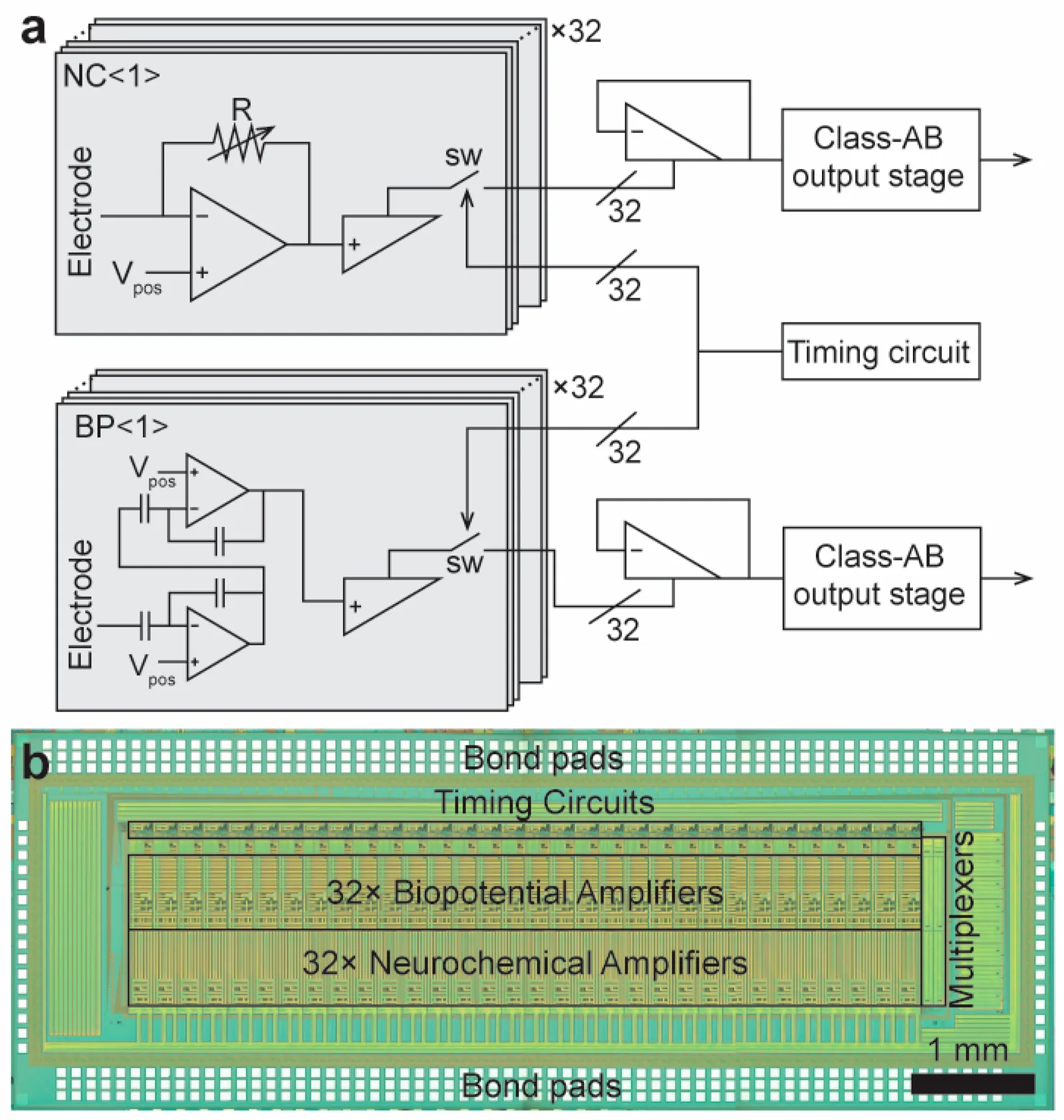
(**a**) A system diagram of the neurochemical and biopotential amplifiers. (**b**) Photomicrograph of the complementary metal-oxide semiconductor (CMOS) chip that includes the neurochemical and biopotential amplifiers, timing circuits, and multiplexers.

The multiplexing circuit is based on a rail-to-rail unity gain amplifier. The multiplexing circuit utilizes a half-shared operational amplifier design in which multiple non-inverting halves of the amplifier (M1N/M1P–M32N/M32P) share a single inverting half of the amplifier (M33N/M33P) (Figure 3) [66]. Each neurochemical and biopotential amplifier output is connected to an input of their respective multiplexing circuit (MUX1–MUX32). Closing each switch (SW1–SW32) connects the non-inverting halves to the inverting half of the amplifier. For instance, when SW1 is closed, the gate of M1N/M1P (MUX1) becomes the positive input, and the gate of the shared inverting transistors (M33N/M33P) is the negative input of the differential pair. By connecting the input (MUX1) with the output of the amplifier (MUXOUT), the pairs form a unity-gain amplifier which outputs the selected input voltage (MUX1). Sequential activation of SW1 to SW32 ensures only one amplifier is being read out, producing a single output (MUXOUT) that multiplexes all the input signals from MUX1 to MUX32. This output connects to external acquisition hardware, requiring the multiplexer to be capable of driving a large capacitive load. A class AB output stage (M38–M43) is employed to handle this requirement. The power consumption of the multiplexing stage is 55.9 µW, and the total power consumption including the class-AB output stage is 479.6 µW. An identical multiplexing circuit is used for both the neurochemical amplifier array and biopotential amplifier array (Figure 4a).

The timing circuit and the multiplexer output interface with a custom data acquisition system. The data acquisition system synchronizes sampling of the analog-to-digital converters (ADCs) with the on-chip timing circuit.

The CMOS chip integrates 32 neurochemical amplifiers, 32 biopotential amplifiers, timing circuits, multiplexers, and bond pads while occupying only a 3 mm × 8.3 mm area, a total area of 24.9 mm^2^ (Figure 4b). The CMOS chip is designed for use with MEAs, enabling the spatially dense recordings critical for resolving small-scale neuronal activities. The integrated design supports large-scale recordings by minimizing interconnect complexity while maintaining high signal fidelity.

## 3. Performance and Characteristics

The gain, bandwidth, and noise performances of the neurochemical and biopotential amplifiers are characterized and discussed.

### 3.1. Characteristics of the Neurochemical Amplifier

All the experiments use a USB-6363 DAQ (National Instruments, Austin, TX, USA) for analog data acquisition. Input signals are generated using either the USB-6363 DAQ or an AFG1062 (Tektronix, Beaverton, OR, USA) function generator. A custom-designed LabVIEW program generates chip control signals. Battery-powered voltage regulators power the chip, minimizing line-frequency noise. A breakout printed circuit board (PCB) is used for straightforward access to each of the chips’ I/O connections.

**Figure 5:**
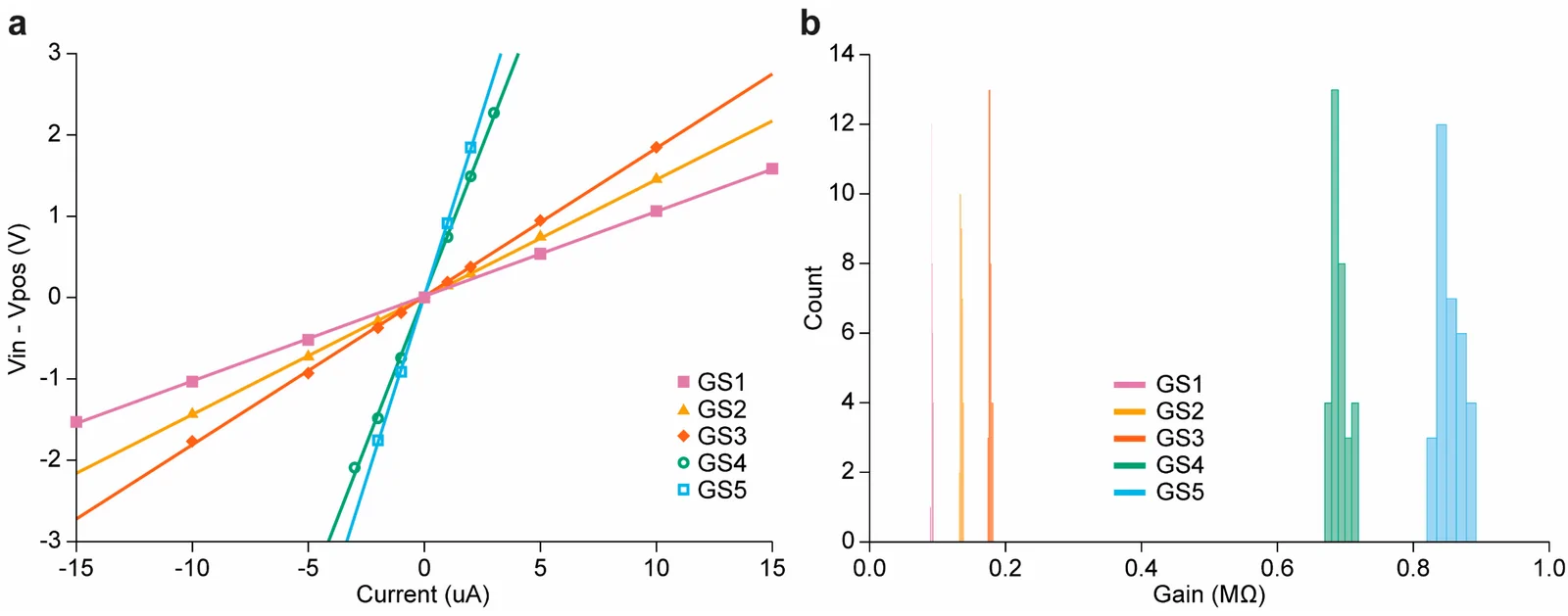
Gain measurements of the neurochemical amplifiers across the chip’s gain settings (GS). (**a**) A representative neurochemical amplifier’s gain for all gain settings. (**b**) A histogram of the measured gain values of all individual amplifiers at each gain setting across the 32 neurochemical amplifiers. From GS1 to GS5, the average (and standard deviation) are respectively: 92.5 ± 1.1 kΩ, 134.5 ± 1.7 kΩ, 177.1 ± 2.1 kΩ, 691.6 ± 13.0 kΩ, and 854.0 ± 18.8 kΩ.

The neurochemical amplifier has variable feedback resistors that can be switched to determine five gain settings (GS). The 32 neurochemical amplifiers have gains of 92.5 ± 1.1 kΩ, 134.5 ± 1.7 kΩ, 177.1 ± 2.1 kΩ, 691.6 ± 13.0 kΩ, and 854.0 ± 18.8 kΩ (mean ± standard deviation) for GS1–GS5, respectively (Figure 5). The amplifiers exhibit highly linear responses with R2 values of each gain setting exceeding 0.998. The dynamic range of the amplifier ranges from ±15 μA (GS1) to as low as ±2 µA (GS5).

**Figure 6:**
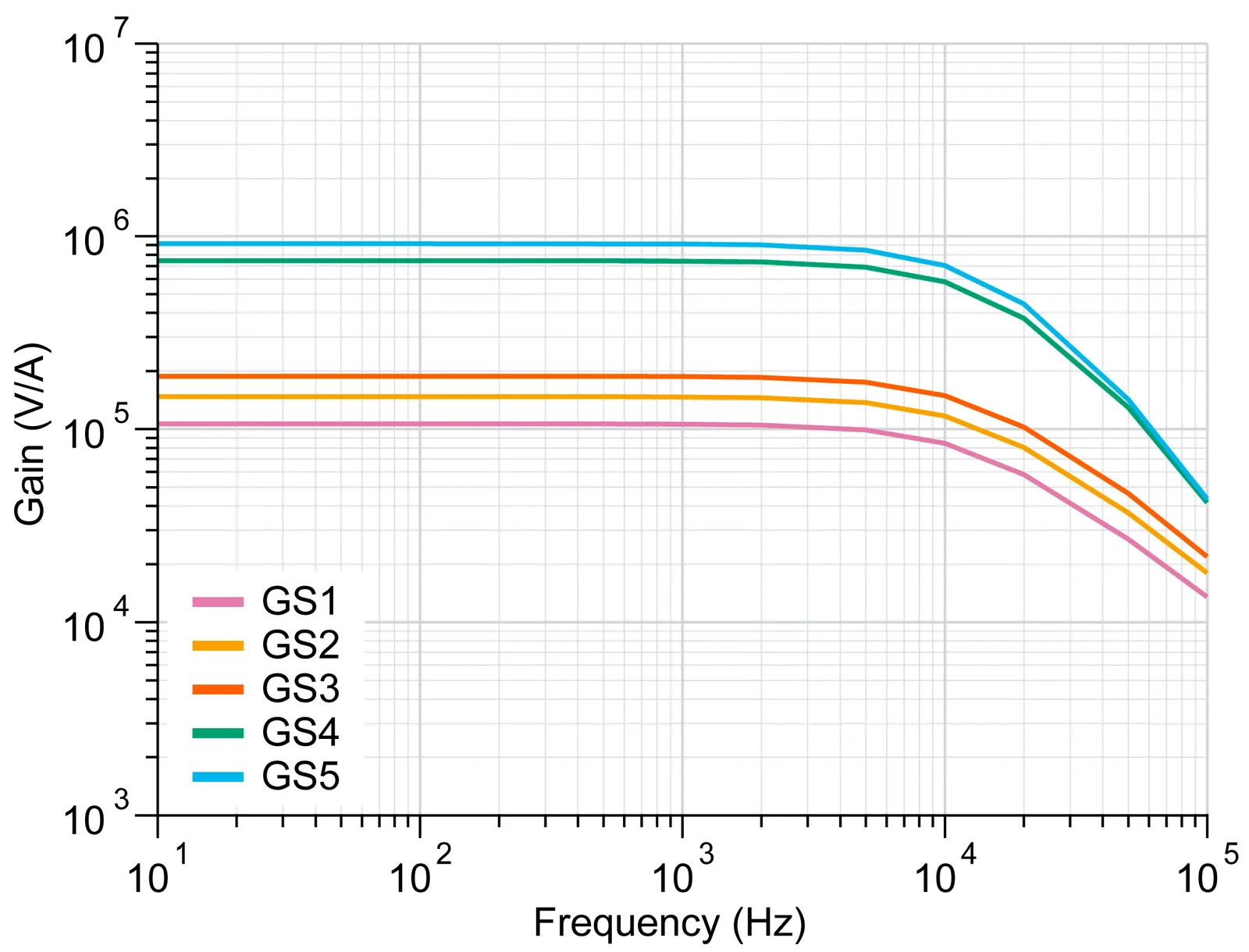
The gain bandwidth measurements of a representative neurochemical amplifier for gain settings (GS) 1 through 5. The neurochemical amplifier bandwidth is 12.6 ± 0.6 kHz (mean ± standard deviation).

The amplifier bandwidth is measured by injecting sinusoidal currents (10 Hz–100 kHz) (Figure 6). A 1 MΩ resistor at the amplifier input converts an applied sine wave voltage into a known input current. The amplifier bandwidth is 12.6 ± 0.6 kHz and is consistent across all gain settings because of the integrated RC low-pass filter at the amplifier output.

**Figure 7:**
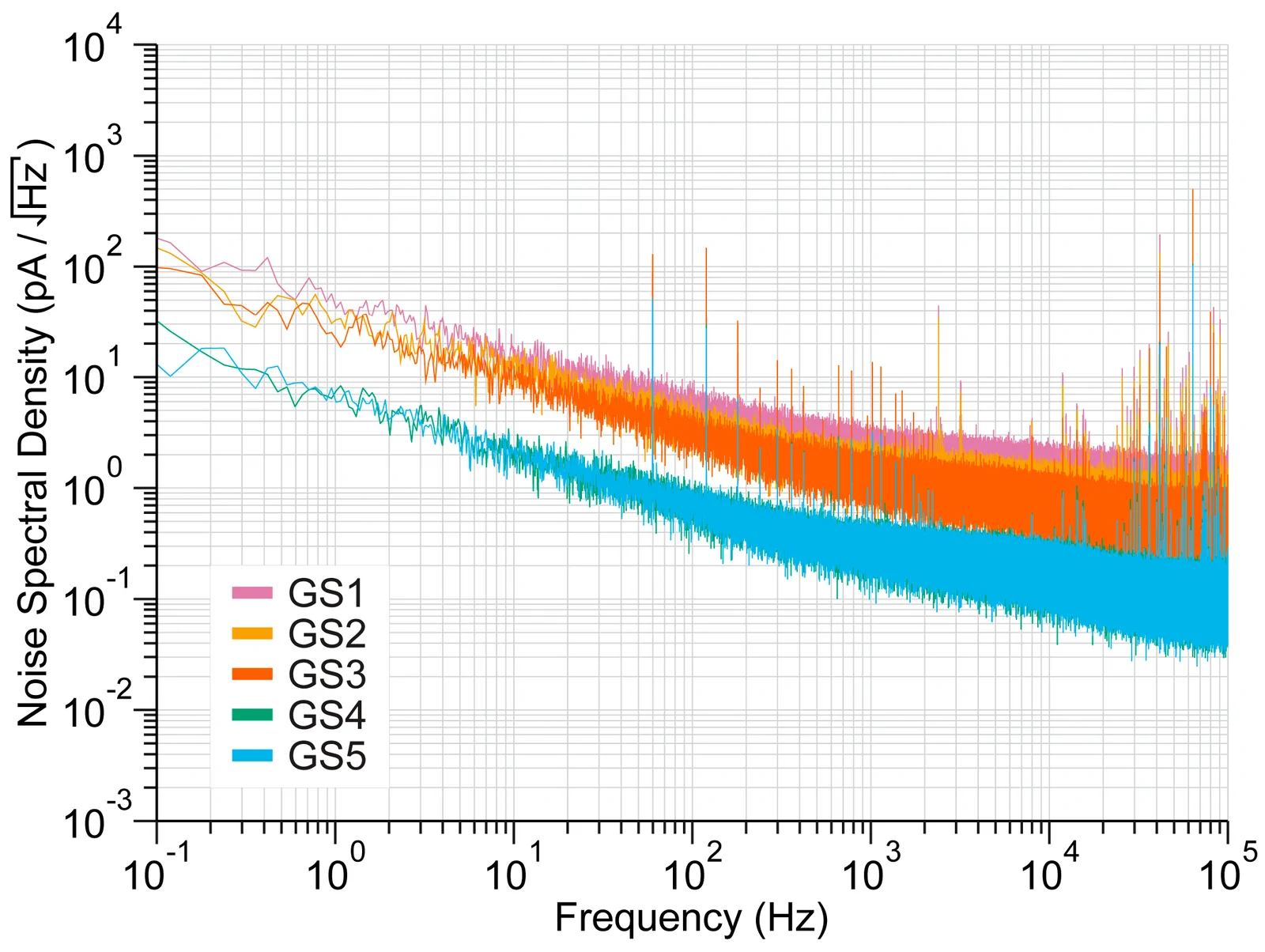
The noise characteristics of a representative neurochemical amplifier across each gain setting (GS). The measured input-referred noise (20 kHz sampling rate) for GS1–GS5, respectively, is 321.9 pA_RMS_, 226.9 pA_RMS_, 195.9 pA_RMS_, 55.6 pA_RMS_, and 46.3 pA_RMS_.

The amplifier array’s input-referred noise, sampled at 20 kHz, for each gain setting are 321.9, 226.9, 195.9, 55.6, and 46.3 pA_RMS_ for GS1–5, respectively, with the highest gain setting (GS5) achieving the lowest noise (46.3 pA_RMS_) (Figure 7). This noise represents the noise contribution of the amplifiers themselves; as this device does not have a dedicated electrode array, this value is separate from noise specific to the electrodes being used.

**Figure 8:**
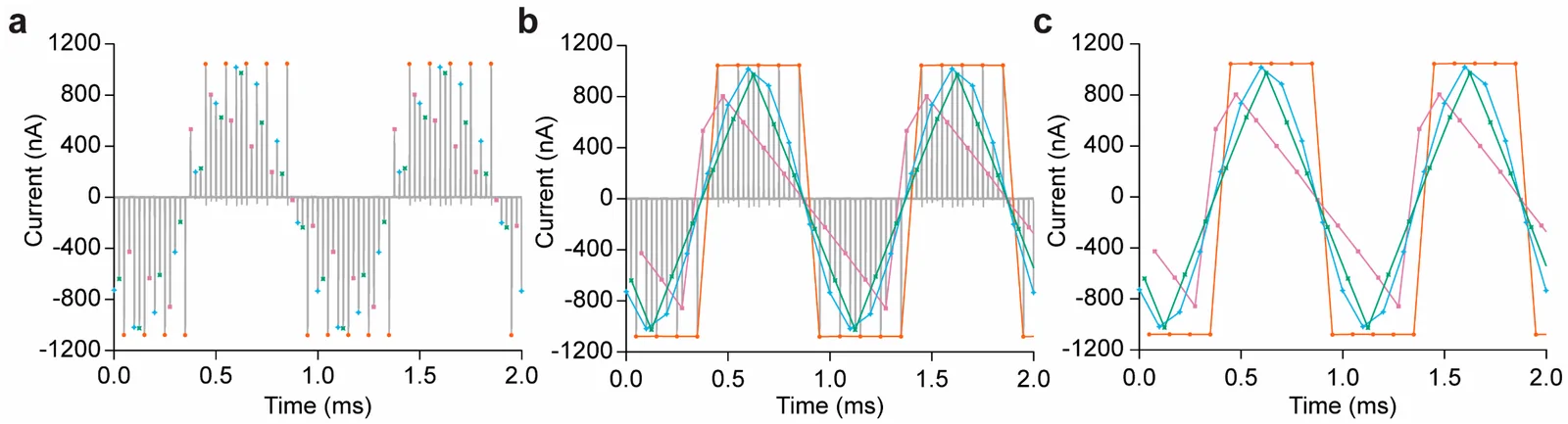
Time-division multiplexing of the neurochemical amplifiers. (**a**) The grey line represents the raw output of the multiplexer with 32 neurochemical amplifiers, four of which are input with current signals. Blue, green, orange, and purple markers correspond to neurochemical amplifiers input with sine, triangle, square, and sawtooth current signals, respectively. (**b**) Demonstration of demultiplexing of each neurochemical amplifier’s output by resampling at a 50 µs interval. (**c**) The demultiplexed signals of the four amplifiers with current signals show clear sine (blue), triangle (green), square (orange), and sawtooth (purple) waves.

A time-multiplexing scheme sequentially routes the outputs of the neurochemical amplifier array to a single common output. To test the multiplexing circuit, four input signals (sine, triangle, square, sawtooth) are passed into the amplifier array; each individual amplifier’s output has been assigned a distinct color (blue, green, orange, and purple) for visualization; each point represents a single measurement from the associated amplifier (Figure 8).

The data show all 32 neurochemical amplifiers’ outputs multiplexed into a single output. Each individual amplifier is sampled at 20 kHz (50 µs period), evenly divided across the 32 amplifiers for readout (~1.56 µs per amplifier). Sequential markers of matching color indicate an interval of 50 µs that occurs between individual amplifier outputs (Figure 8a). For demultiplexing, the raw MUXOUT output is resampled at a 50 µs interval (Figure 8b). Isolating the individual demultiplexed amplifiers from the multiplexed MUXOUT output yields the sine, triangle, square, and sawtooth input signals (Figure 8c).

### 3.2. Characteristics of the Biopotential Amplifier

The gain, bandwidth, and noise of the biopotential amplifier array are characterized in this section. The gain and bandwidth are determined using sine-wave voltage inputs. Voltage dividers (10 kΩ/100kΩ) are placed between the function generator’s output and the chip’s inputs to limit the input signal to the µV–mV range. The function generator produces sine-wave inputs ranging from 0.05 Hz to 50 kHz.

**Figure 9:**
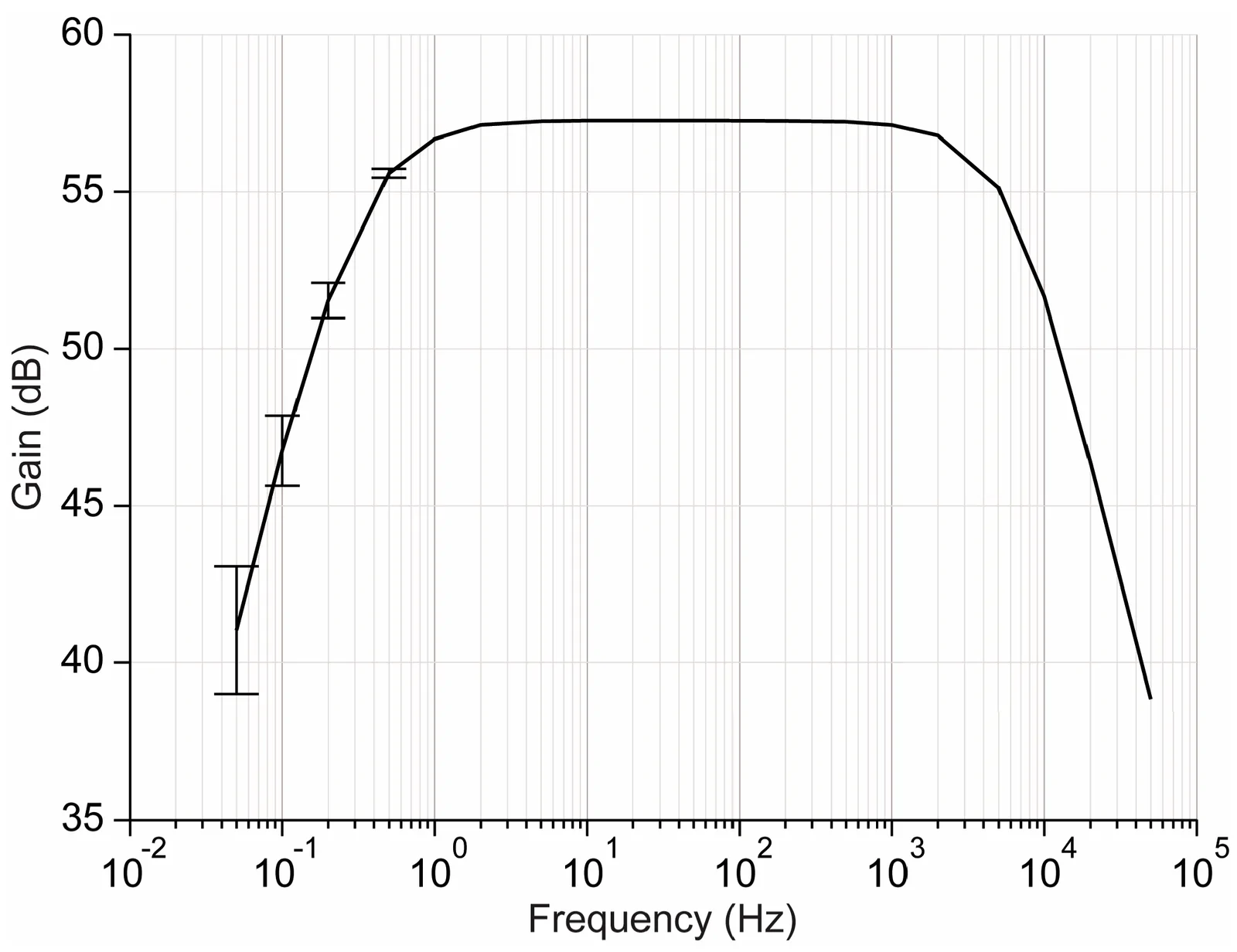
The bandwidth measurement of the biopotential amplifiers. Each amplifier is measured from 0.05 Hz to 50 kHz. The bandwidth is measured to be 6.2 ± 0.1 kHz. Error bars indicate standard deviation, with standard deviations less than 0.2% of the maximum value removed for visual clarity.

**Figure 10:**
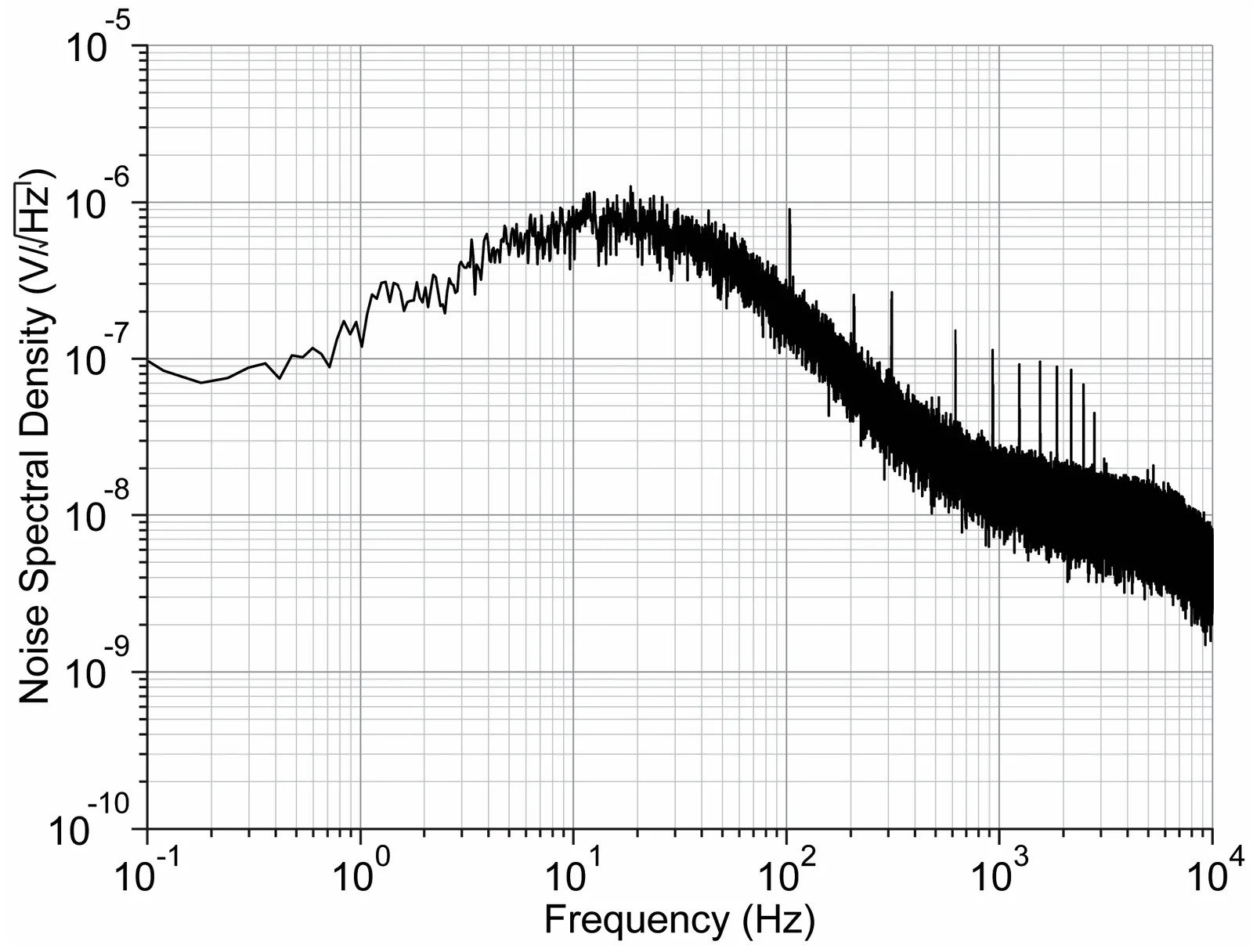
The noise characteristics of a representative biopotential amplifier. The noise level is measured to be 6.74 ± 0.92 µV_RMS_ at a 20 kHz sampling rate.

The biopotential amplifier array has an average passband gain of 57.10 ± 0.04 dB, a low-frequency cutoff of 0.4 Hz, and a bandwidth of 6.2 kHz (Figure 9). The passband is adequate for typical local field potentials that range from 0.5 Hz to 200 Hz and action potentials from 300 Hz to 5 kHz. The input-referred noise of the array is 6.74 ± 0.92 µV_RMS_ using a sampling rate of 20 kHz (Figure 10).

**Figure 11:**
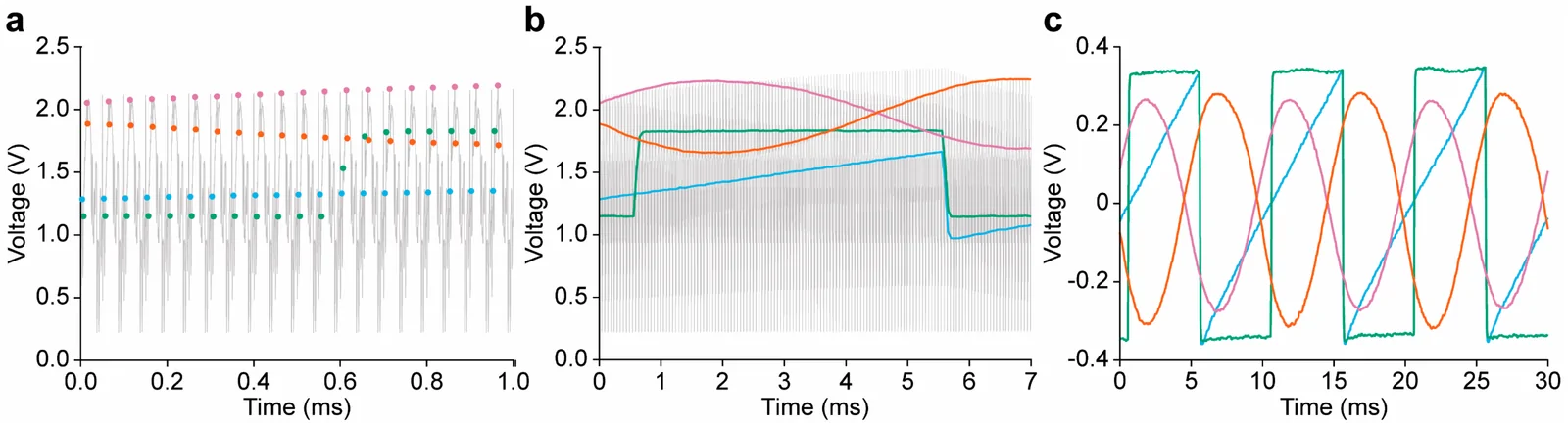
Time-division multiplexing of the biopotential amplifier. (**a**) The raw output (shown in grey line) multiplexes 32 individual biopotential amplifiers’ outputs. Blue, green, orange, and purple markers indicate biopotential amplifiers’ inputs with sawtooth, square, sine, and phase-shifted sine voltage signals, respectively. (**b**) By resampling the raw output at a 50 µs interval, each biopotential amplifier’s output can be demultiplexed. (**c**) The demultiplexed signals from the four amplifiers show clear sawtooth (blue), square (green), sine (orange), and phase-shifted sine (purple) waves.

The multiplexing scheme for the biopotential amplifier array is identical to that of the neurochemical amplifier array. All 32 biopotential amplifier outputs are multiplexed into a single output through time-division multiplexing (Figure 11). The multiplexed output of the individual amplifier signals is separated by time-division (Figure 11a,b) and reassembled digitally (Figure 11c).

## 4. Silicon-Based Microelectrode Array Probe Fabrication

**Figure 12:**
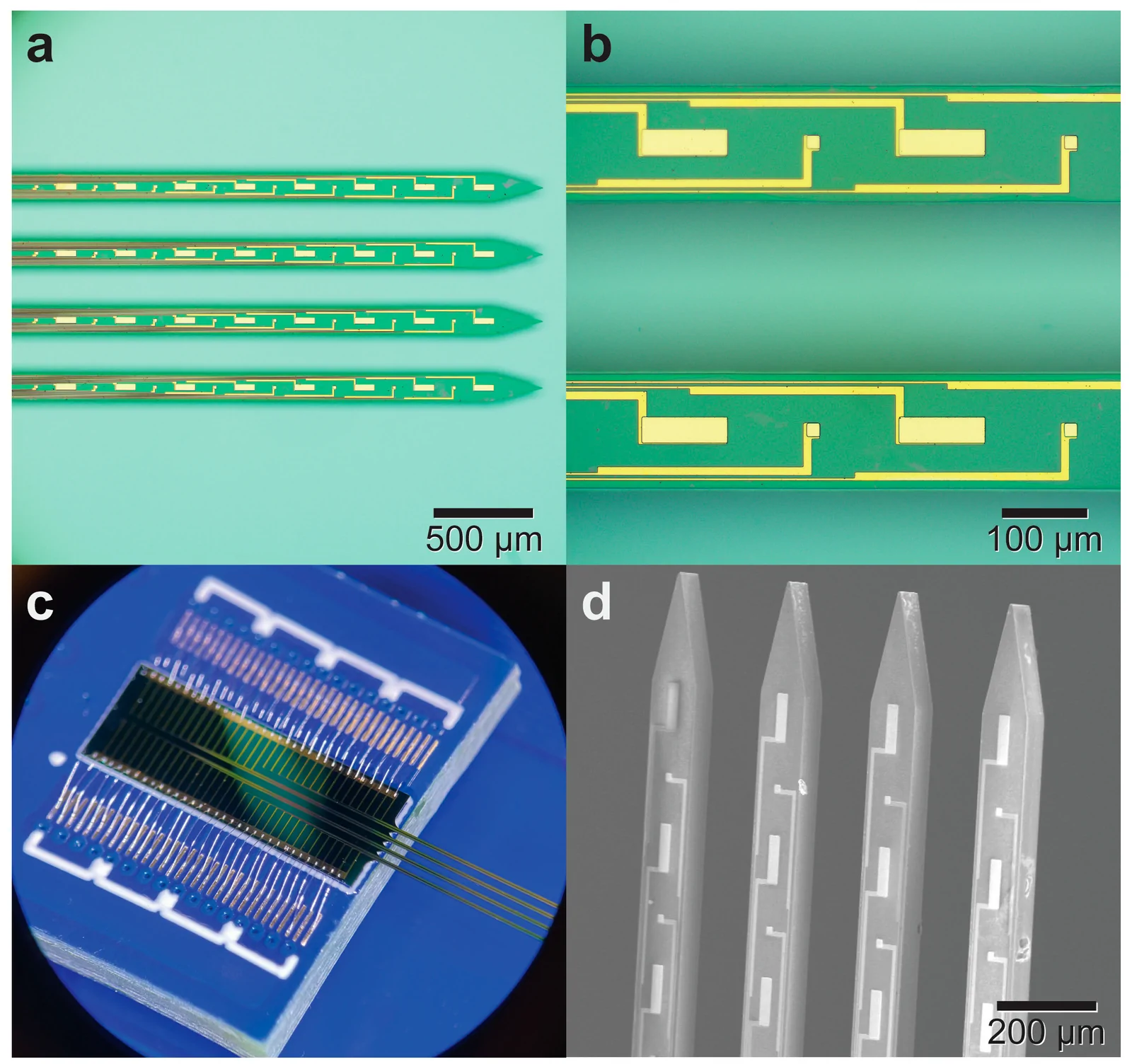
Silicon-based microelectrode array probe. (**a**) Microphotograph of the probe with four parallel shanks. Each shank contains eight biopotential and eight neurochemical electrodes, for a total of 64 electrodes across the four shanks. (**b**) The neurochemical electrodes are 100 µm × 30 µm, and the biopotential electrodes are 15 µm × 15 µm. (**c**) Microphotograph of the MEA probe wire-bonded to a PCB. (**d**) Scanning electron microscope image of the MEA probe.

The silicon-based microelectrode array probe is fabricated from a 4-inch, double-side polished silicon wafer with a thickness of 300 µm. A 200 nm layer of silicon dioxide is deposited on the wafer to electrically isolate the silicon substrate from subsequent metal layers. Using photolithography and lift-off, a 50 nm titanium layer and a 200 nm gold layer are deposited and patterned. A 300 nm layer of silicon dioxide is deposited to form a second insulation layer. Small openings are created to expose the gold electrodes. Probe singulation is achieved using deep reactive-ion etching (60 µm) on the top to define the probe shape, which is followed by ~250 µm of backside etching. Each probe integrates 32 microelectrodes for neurochemical sensing and 32 microelectrodes for biopotential recordings distributed equally into four shanks (Figure 12). For packaging, the probe is wire-bonded to a custom PCB and the bonds are insulated using black epoxy (Resinlab EP965).

## 5. Catecholamine Recordings Using a Microelectrode Array

To validate the neurochemical amplifiers’ catecholamine measurement capabilities, the 32-microelectrode silicon-based probe is connected to the neural amplifier for in vitro testing. Dopamine is introduced to the probe’s MEA using a flow cell setup, during which FSCV recordings are taken.

### 5.1. Flow Cell Experiment

An in vitro FSCV microelectrode flow cell (NEC-FLOW-1, Pine Research, Durham, NC, USA) was used to deliver catecholamine solutions to the neurochemical sensing array. The flow cell operates by gravity feed, with the sample flowing toward the electrode array. An Ag|AgCl reference electrode, shared by the biopotential and neurochemical amplifiers, was biased to 2.2 V to maintain the CMOS inputs within the 0–5 V supply range. The FSCV waveform was applied relative to the reference electrode, with a potential range of −0.4 to 0.7 V. PBS was perfused through the flow cell at ~1 mL/min, and 1 µM dopamine (DA) or norepinephrine (NE) solutions were introduced using a switch valve controlled by a syringe pump. In this proof-of-concept implementation, the neurochemical and biopotential amplifiers share a reference electrode.

### 5.2. Fast-Scan Cyclic Voltammetry Recordings

**Figure 13:**
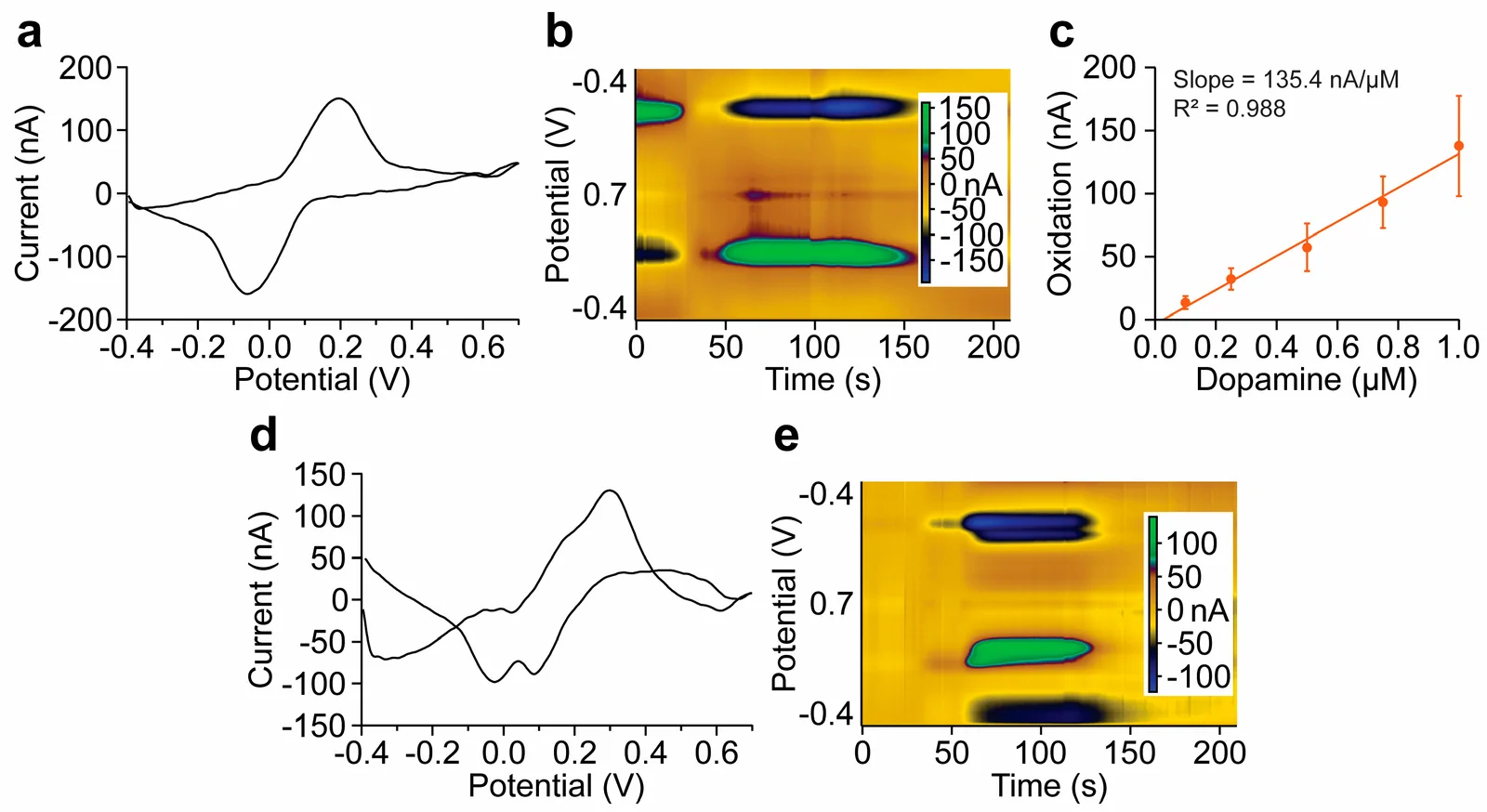
Catecholamine measurements using the microelectrode array probe placed within a flow cell using FSCV. (**a**) DA recording with an oxidation peak of 151.1 nA and a reduction peak of −159.7 nA. (**b**) Color plot displaying current amplitude (pixel color) across the scanning potential (*y*-axis) over time (*x*-axis) for the DA measurement. (**c**) Calibration curve for dopamine, plotting oxidation peak versus dopamine concentration. (**d**) NE recording with an oxidation peak of 130.7 nA and a reduction peak of −98.3 nA. (**e**) Color plot for the NE measurement.

The neurochemical amplifiers share a common V_pos_ connection, which is used to apply the FSCV ramp, sweeping the potential from 1.8 to 2.9 V (−0.4 to 0.7 V versus Ag|AgCl held at 2.2 V) at 100 V/s. The ramp repeats every 100 ms with a resting potential of −0.4 V, while PBS is perfused at ~1 mL/min. A five-second baseline recording is collected before catecholamine injection for background subtraction. After injecting 1 µM of dopamine (DA) or norepinephrine (NE), the background-subtracted voltammograms show distinct oxidation and reduction peaks (Figure 13a,d), consistent with prior FSCV measurements [11,13,21]. A 1 µM concentration is selected as a higher concentration test condition for characterization of the front-end. Time-averaged extracellular dopamine concentrations in the rat nucleus accumbens are typically reported around 20–30 nM, with transients reaching several hundred nanomolar [67,68,69]. The DA recording has oxidation and reduction peaks of 151.1 and −159.7 nA, respectively, versus 130.7 and −98.3 nA for NE. The measured DA peak currents match the calibrated response for the tested concentrations (Figure 13c). The similarity between the DA and NE voltamograms is expected given their related chemical structures.

The measured noise depends on the input capacitance of the microelectrode. For the 30 × 100 µm^2^ gold microelectrode submerged in PBS, the measured noise is ~2.2 nA at the lowest gain setting and ~300 pA at the highest gain setting.

The microelectrode array-based DA/NE recordings validate the neurochemical front-end for catecholamine detection. The recorded cyclic voltammograms are plotted as 2D color maps to visualize DA/NE oxidation and reduction over time (Figure 13b,e). In the color plots, each pixel increment along the *x*-axis represents a 100 ms interval (FSCV measurements are typically taken at a rate of 10 Hz), each vertical line of pixels represents a single FSCV measurement, in which the color represents the measured current at the potential value occurring during the stimulation, represented along the *y*-axis. The flow cell setup provides a controlled introduction of DA/NE to the probe over 50 to 150 s, which produces a significant signal with strong oxidation and reduction currents (Figure 13b,d).

## 6. Biopotential Recordings

The amplifier array’s biopotential recording capabilities are demonstrated using three experimental setups. The first setup (1) directly injects synthesized neural spikes into the amplifier’s input. The second setup (2) places a stimulation electrode and neural probe into a glass beaker of PBS, where neural spikes are injected into the stimulation electrode and recorded through the neural probe. The third setup (3) places the stimulation electrode and neural probe into a phantom brain (agarose gel) and injects the neural spikes for recording. For each of the setups, the synthesized neural spikes are generated using an NI DAQ; the highest DAC resolution is used and the dynamic range is limited to 1V. A voltage divider is set up to reduce the amplitude to simulate in vivo levels. All recordings are processed with a 60 Hz notch filter.

**Figure 14:**
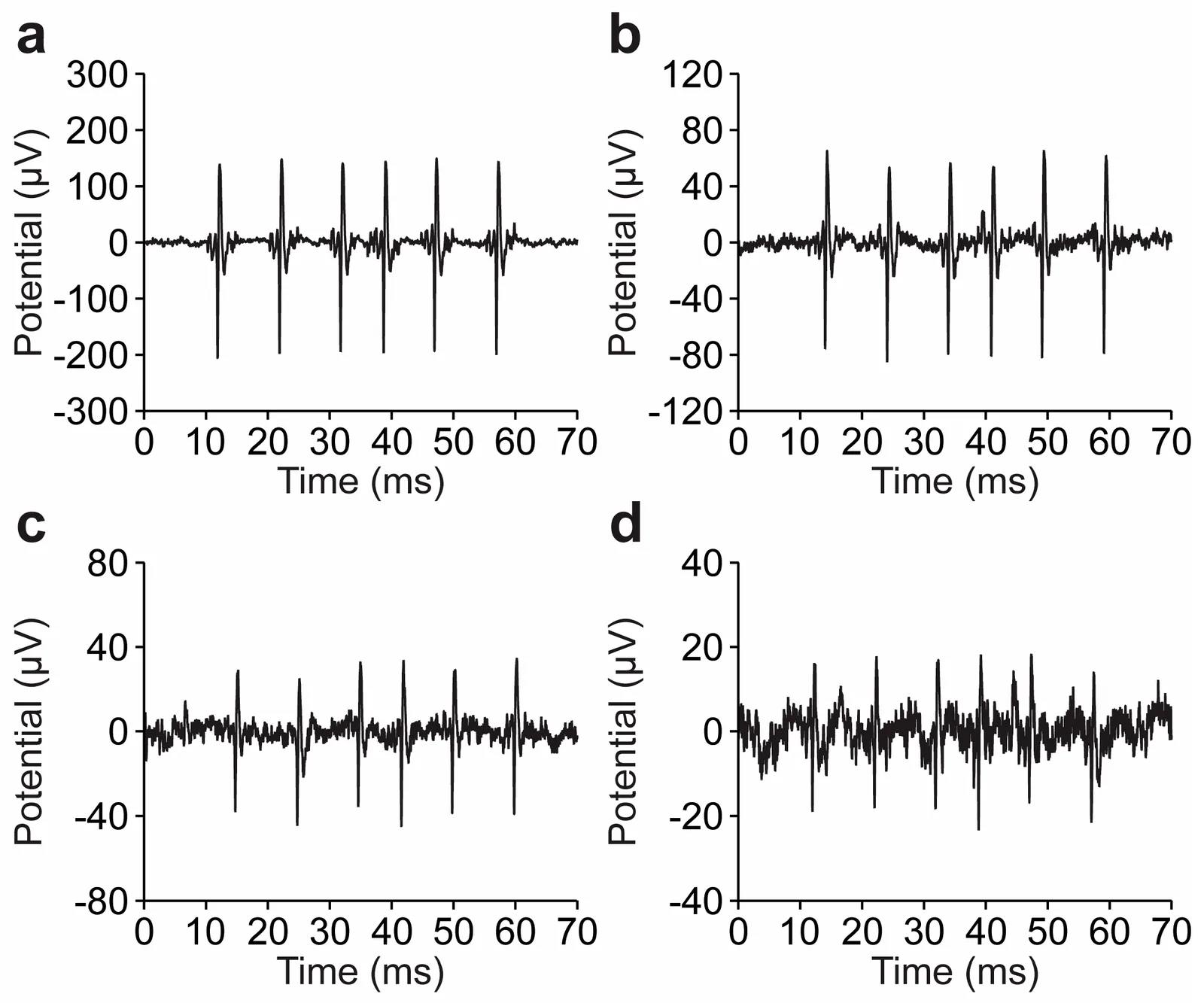
Biopotential recordings with signals applied directly to the amplifier’s input at various amplitudes. Measurements of (**a**) 340 µV, (**b**) 130 µV, (**c**) 75 µV, (**d**) 40 µV peak-to-peak input signals.

For the direct injection setup (1), the measured data (Figure 14) show voltage inputs with peak-to-peak magnitudes from 340 µV to 40 µV, with each set of neural spikes remaining observable across each magnitude. These measurements validate the biopotential amplifier’s ability to record small amplitudes (10 s of µV).

**Figure 15:**
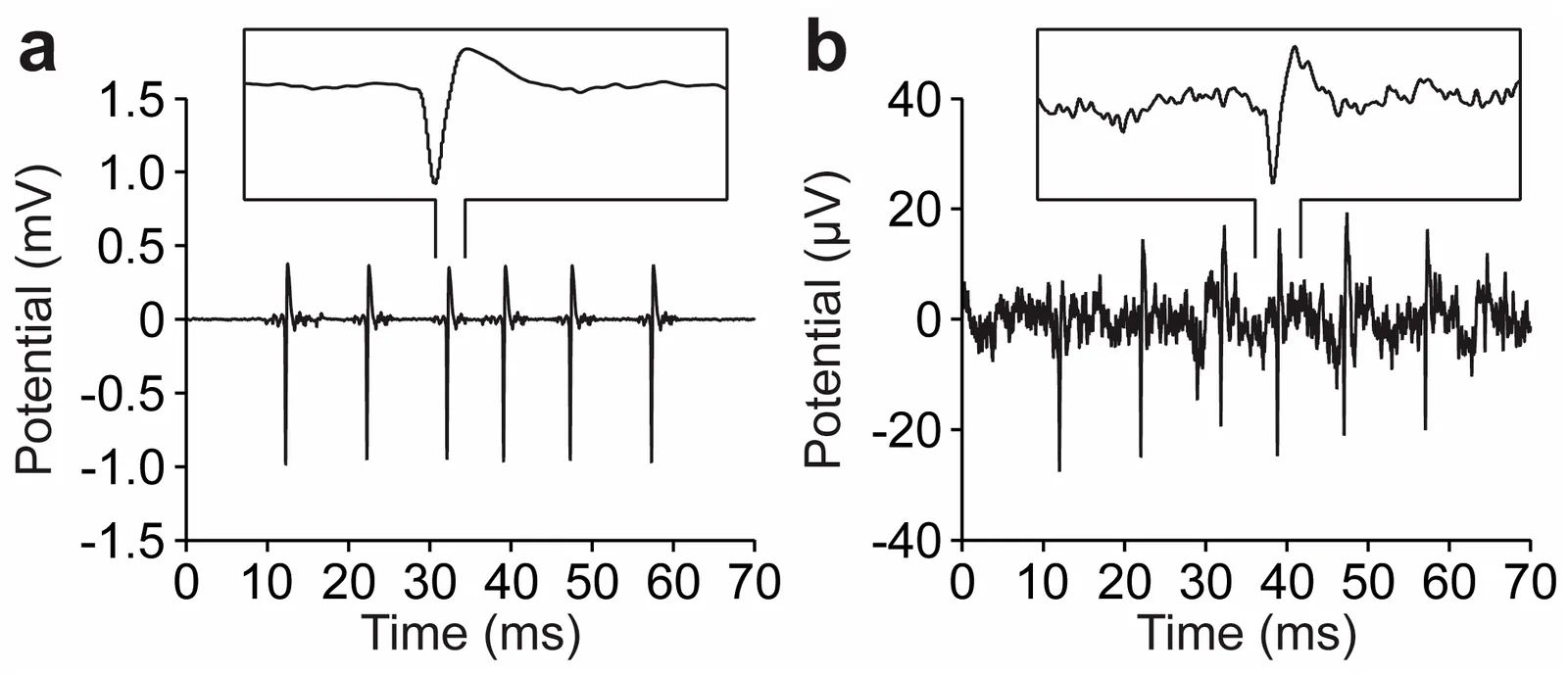
A biopotential stimulation recording with the stimulation electrode and neural probe within a PBS solution. Measurements of (**a**) 1.33 mV and (**b**) 40 µV peak-to-peak neural spikes are shown.

For the second setup (2), we prepared 30 mL of PBS solution using 10× PBS pH 7.4 (Gibco) stock solution in a 50 mL beaker. A stimulation electrode (a bare platinum/iridium wire of 254 µm diameter, P1 Technologies), reference electrode (Ag|AgCl), and our neural probe are lowered 1 cm into the PBS solution; a 1 cm gap separates each from one another. The stimulation electrode is injected with a series of neural spikes with specific peak-to-peak voltages; the length of the bare stimulation wire results in a low electrode–electrolytic interface impedance. These spikes are recorded through the neural probe’s electrodes, which are connected to the biopotential amplifier. In an in vivo experiment, the recorded signals would be within the range of tens to low hundreds of microvolts. A large peak-to-peak signal of 1.33 mV is successfully recorded, as well as small 40 µV signals in the PBS solution, which demonstrate the device’s ability to capture larger biopotentials without distortion or saturation, in addition to small signals (Figure 15).

**Figure 16:**
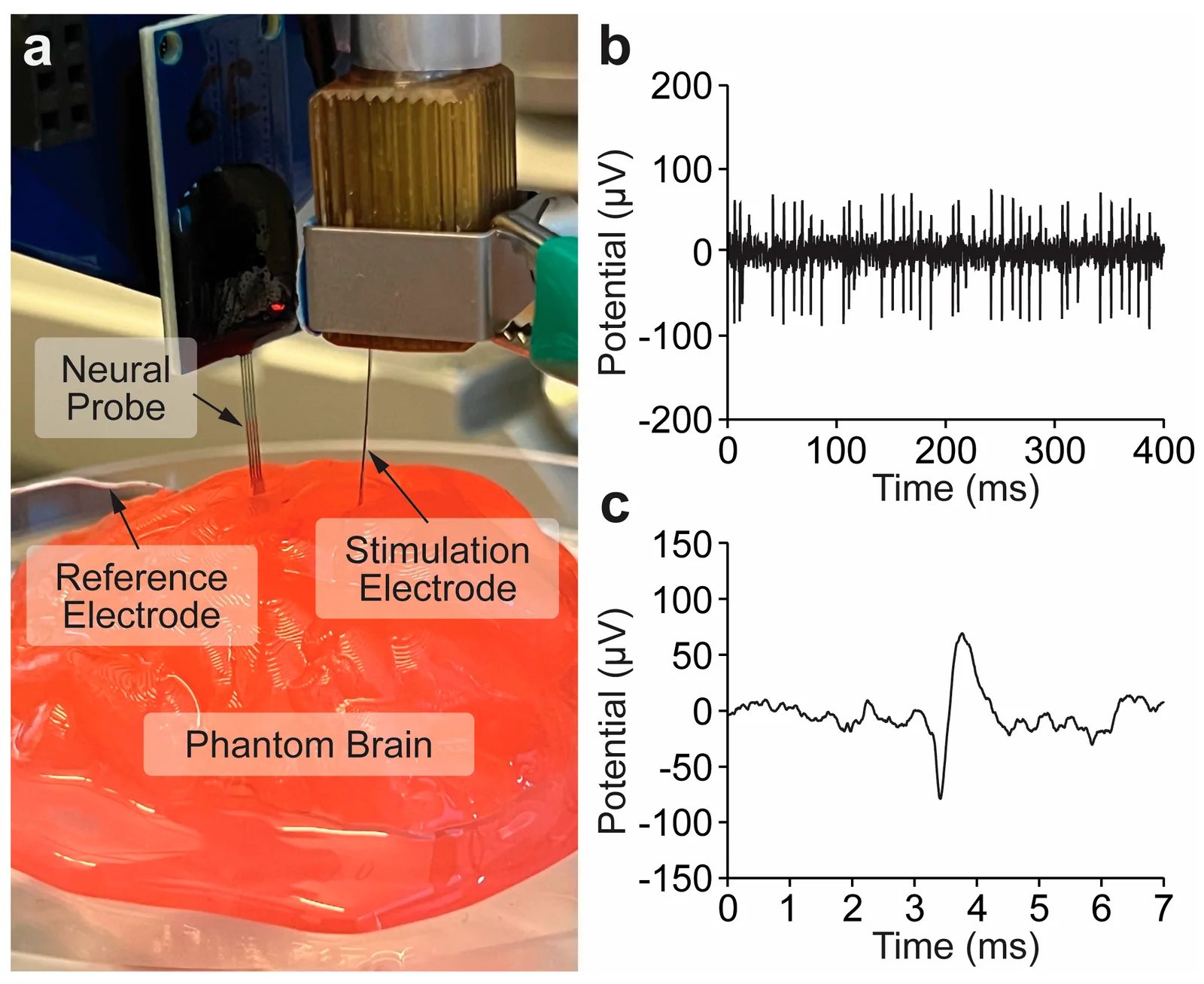
Biopotential recordings using an agarose gel “phantom brain”. (**a**) Photograph of the agarose gel experimental setup. (**b**) Measurement of 130 µV peak-to-peak neural spike and (**c**) a single spike from the series.

In the third experimental setup (3), a 2% (*w*/*w*) agarose gel (VWR Chemicals, Radnor, PA, USA) serves as a phantom brain for the experiment. The reference electrode, stimulation electrode and neural probe are placed into the phantom brain 1 cm apart (Figure 16a). A series of neural spikes is applied to the stimulation electrode with peak-to-peak voltages varying between trials. A 130 µV peak-to-peak voltage neural spike series is presented (Figure 16b). A short time span from this series has been chosen to display an isolated neural spike (Figure 16c). For the three testing setups, the microelectrode array-based probe is mounted on a breakout board for connections (Figure 16a). In practice, a dedicated headstage further reduces noise and enables a low-noise recording setup.

## 7. Discussion and Conclusions

This work presents a CMOS platform integrating 32 neurochemical and 32 biopotential amplifiers for scalable multimodal neural recording. Compared with existing dual-mode front-ends, the proposed architecture provides a neurochemical amplifier with a wide dynamic range and selectable gain to accommodate electrodes with different dimensions and materials (Table 1). The neurochemical amplifier is further compared with recent neurochemical CMOS ICs featuring wide dynamic ranges (Table 2).

Simultaneous neurochemical and biopotential measurements can provide complementary information about neurotransmitter signaling and electrophysiological activity, with potential applications in neuroscience and neuroprosthetics. Future studies will investigate the crosstalk between the neurochemical amplifiers and biopotential amplifiers in conjuction with a microelectrode-array-based probe in in vivo systems.

## Figures and Tables

**Table 1:** State-of-the-art dual-mode (electrochemical/biopotential) sensing front-ends.

	2015 [52]	2017 [53]	2018 [54]	2020 [55,56]	2023 [57]	This Work
Technology Node (nm)	180	180	130	180	350	350
Die Size (mm^2^)	5 × 2.65	12 × 8.9	3 × 1.85	~10 × ~20	5 × 5	3 × 8.3
Operation Voltage (V)	1.8	-	3	3.6	3.3	5
Application	In Vitro	In Vitro	In Vitro	In Vitro	In Vitro	In Vivo
Electrochemical						
Parallel Channels (#)	100	28	4	4096	256	32
Size Per Channel (µm^2^)	30,000	40,000	8000	25,000	3070	120,000
Sampling Rate (kHz)	20	20	100	9.4	40	20
Bandwidth (kHz)	0.11–10	16	0.7	4.7	10	12.6
Noise	93 pA (FSCV)	120–200 pA	56 pA	~1 pA	4.51 pA	43–320 pA *
Dynamic Range	±50 nA	±1 µA	-	-	±2–6 µA	±2–15 µA *
Gain	0.011–2.38 GΩ	2.7–3.2 MΩ	129 kΩ	~700 MΩ	0.625–2.08 MΩ	92.5–854 kΩ *
Power (µW)	12.1	178	-	249	16	30–432 *
Biopotential						
Parallel Channels (#)	100	2048	1024	4096	256	32
Size Per Channel (µm^2^)	30,000	-	-	25,000	11,080	120,000
Sampling Rate (kHz)	20	20	-	9.4	40	20
Bandwidth (kHz)	10	0.3–10	0.3–6	4.7	0.0002–10	6.2
Noise (µV)	4.07	2.4	7.1	20	24.9	6.7
Power (µW)	9.1	16	-	225	26	97

* Based on variable gain settings.

**Table 2:** State-of-the-art large dynamic range neurochemical CMOS ICs.

	2017 [20]	2020 [26]	2021 [27]	2022 [29]	2023 [57]	2024 [28]	2024 [31]	This Work
Technology Node (nm)	65	180	180	180	350	350	180	350
Supply Voltage (V)	1.8	2.5	1.2–1.5	3.3	3.3	4	3.3/1.8	5
Number of Channels (#)	4	1	1	1	256	128	2	32
Channel Size (mm^2^)	0.075	0.06	-	0.06	0.00307	0.0176	-	0.12
Total Die Area (mm^2^)	-	-	3.17	0.256	25	4.18	6.4	24.9
Input Current Range (µA)	±2.56	±1	−7/+10	±0.375	±4.8	±5	±10 pA–1 mA	±2–15 *
Input Referred Noise (pA_RMS_)	20	710	87	26.5	4.51	220	12.88	46.3–321.9 *
Bandwidth (kHz)	1	5	50	5	10.3	40–122	10	12.6
Power Consumption	3.1 µW/Ch	14.1 µW/Ch	1.77 µW/Ch	3.7 µW/Ch	16 µW/Ch	4.4 µW/Ch	4.36 mW/Chip	30–432 µW/Ch *

* Based on variable gain settings.

## Data Availability

The raw data supporting the conclusions of this article will be made available by the authors on request.

## Abbreviations

The following abbreviations are used in this manuscript:

FSCV	Fast-Scan Cyclic Voltammetry
CMOS	Complementary Metal-Oxide-Semiconductor
AFE	Analog Front-End
RMS	Root Mean Square
MEA	MicroElectrode Array
IC	Integrated Circuit
nMOS	N-type Metal-Oxide-Semiconductor
pMOS	P-type Metal-Oxide-Semiconductor
LFP	Local Field Potential
AP	Action Potential
ADC	Analog-to-Digital Converter
PCB	Printed Circuit Board
GS	Gain Setting
PBS	Phosphate-Buffered Saline
DA	Dopamine
NE	Norepinephrine
